# Amyloid-β induces synaptic dysfunction through G protein-gated inwardly rectifying potassium channels in the fimbria-CA3 hippocampal synapse

**DOI:** 10.3389/fncel.2013.00117

**Published:** 2013-07-25

**Authors:** Mauricio O. Nava-Mesa, Lydia Jiménez-Díaz, Javier Yajeya, Juan D. Navarro-Lopez

**Affiliations:** ^1^Laboratorio Neurofisiología y Comportamiento, Facultad de Medicina de Ciudad Real, Universidad de Castilla-La ManchaCiudad Real, Spain; ^2^Department of Fisiología y Farmacología, Universidad de SalamancaSalamanca, Spain

**Keywords:** septohippocampal system, fimbria-CA3 synapse, amyloid-β_25–35_ peptide, GABA_B_, GirK channels, Alzheimer's disease, brain slices, intracellular recordings

## Abstract

Last evidences suggest that, in Alzheimer's disease (AD) early stage, Amyloid-β (Aβ) peptide induces an imbalance between excitatory and inhibitory neurotransmission systems resulting in the functional impairment of neural networks. Such alterations are particularly important in the septohippocampal system where learning and memory processes take place depending on accurate oscillatory activity tuned at fimbria-CA3 synapse. Here, the acute effects of Aβ on CA3 pyramidal neurons and their synaptic activation from septal part of the fimbria were studied in rats. A triphasic postsynaptic response defined by an excitatory potential (EPSP) followed by both early and late inhibitory potentials (IPSP) was evoked. The EPSP was glutamatergic acting on ionotropic receptors. The early IPSP was blocked by GABA_A_ antagonists whereas the late IPSP was removed by GABA_B_ antagonists. Aβ perfusion induced recorded cells to depolarize, increase their input resistance and decrease the late IPSP. Aβ action mechanism was localized at postsynaptic level and most likely linked to GABA_B_-related ion channels conductance decrease. In addition, it was found that the specific pharmacological modulation of the GABA_B_ receptor effector, G-protein-coupled inward rectifier potassium (GirK) channels, mimicked all Aβ effects previously described. Thus, our findings suggest that Aβ altering GirK channels conductance in CA3 pyramidal neurons might have a key role in the septohippocampal activity dysfunction observed in AD.

## Introduction

Being still lack of effective treatments for Alzheimer's disease (AD), current research efforts have focused on finding the relationships between amyloid-β peptide (Aβ) functions and toxic mechanisms to understand the development of AD (Huang and Mucke, 2012). Memory deficits and disorientation appear as the first symptoms of AD (McKhann et al., 1984; Swanberg et al., 2004) and, among the different regions early affected, damages found in septum and hippocampus could explain these cognitive deficits (Moreno et al., 2007; Palop et al., 2007; Villette et al., 2010; Rubio et al., 2012). Both structures are reciprocally interconnected through fimbria/fornix, and are functionally coupled to form the septohippocampal system (Bland and Colom, 1993), which is critical in generating certain oscillatory activity, such as *theta* rhythm, necessary for fundamental processes in learning and memory (Stewart and Fox, 1990; Bland and Oddie, 2001; Buzsaki, 2002; Sotty et al., 2003; Colom, 2006; Colom et al., 2010; Rubio et al., 2012). *Theta* oscillation coordinates septohippocampal network and depends on interconnections, which include well known cholinergic and GABAergic (Lynch et al., 1977; Kohler et al., 1984; Bland and Colom, 1993) as well as glutamatergic (Sotty et al., 2003; Huh et al., 2010) projections.

In animal models of AD, septohippocampal network dysfunction has extensively been reported (Colom, 2006; Palop and Mucke, 2010; Peña et al., 2010; Villette et al., 2010, 2012; Rubio et al., 2012; Verret et al., 2012). At the synaptic level, dysfunction induced by Aβ on inhibitory neurotransmission causes aberrant patterns of activity in its associated neural circuits, destabilizes neuronal networks and impairs oscillatory activity. This scenario, ultimately, seems to be responsible for the early alteration of the processes implicated in learning and memory tasks observed in AD patients (Palop and Mucke, 2010; Huang and Mucke, 2012). However, the specific mechanisms involving inhibitory neurotransmission at the molecular level, synaptic circuits or systems that consistently explain Aβ neurotoxic effects and associated neurological deficits remain unknown.

γ-aminobutyric acid (GABA) is the main inhibitory neurotransmitter in the mammalian central nervous system and is involved in the regulation of many physiological processes. GABA mediates the inhibitory neurotransmission and accordingly, regulates excitatory activity preventing hyperexcitation, actions especially relevant to maintain neural network stability and oscillatory activity (Palop and Mucke, 2010). GABA metabotropic type receptors (GABA_B_) are coupled to intracellular signal transduction mechanisms via G proteins (Mott and Lewis, 1994; Kaupmann et al., 1998) and mediate slow and prolonged synaptic inhibition mainly by postsynaptic G protein-coupled activated inwardly-rectifying potassium (GirK) channels (Luscher et al., 1997; Kaupmann et al., 1998). Thus, GirK channels act as key players in the control of cellular and network excitability by modulating synaptic activity (Lujan and Ciruela, 2012).

In this study, we aimed to characterize Aβ effects on septohippocampal fimbria/CA3 synapsis. To address this question, we used an *in vitro* preparation taking advantage of the specific septo-hippocampal projection to CA3 pyramidal neurons, and evoked a characteristic complex synaptic response in CA3 recorded neurons by stimulating the septal part of the fimbria. For the first time, we provide evidence that Aβ decreased GABA_B_ neurotransmission through altering GirK channel conductance.

## Materials and methods

### Animals

Experiments were carried out on male and female rats (80–100 g) raised in the Salamanca University Animal House (Salamanca, Spain). All animal procedures were reviewed and approved by the Ethical Committee for Use of Laboratory Animals of the University of Salamanca and University of Castilla-La Mancha, and followed the European Communities Council (86/609/EEC).

### Preparation of slices

Animals were deeply anesthetized with halothane and decapitated. The brain was excised and rapidly immersed in oxygenated ice-cold (4–6°C) artificial cerebrospinal fluid (ACSF), with sucrose (234 mM) replacing the NaCl (117 mM) to maintain osmolarity. In order to preserve the optimal connectivity from fimbria fibers on CA3 pyramidal neurons (Gloveli et al., 2005; Bischofberger et al., 2006), horizontal slices containing the septal part of the fimbria, i.e., lateral fimbria (Alonso and Kohler, 1984; Amaral and Lavenex, 2007), and the hippocampus (350 μm-thick) were cut in cold oxygenated Ringer solution using a vibratome (Leica VT 1000S, Wetzlar, Germany) and placed in an incubation chamber, where they were maintained for at least 2 h at room temperature (22°C) before the recordings. Further details of this *in vitro* preparation have been described elsewhere (Yajeya et al., 2000).

### Sharp electrode recordings

For recordings, a single septohippocampal slice was transferred to an interface recording chamber (BSC-HT and BSC-BU; Harvard Apparatus, Holliston, US) and perfused continuously with ACSF comprising (in mM) 117 NaCl, 4.7 KCl, 2.5 CaCl_2_, 1.2 MgCl_2_, 25 NaHCO_3_, 1.2 NaH_2_PO_4_, and 11 glucose. The ACSF was bubbled with carbogen gas (95%O_2_–5%CO_2_) and maintained at room temperature during the recordings.

Intracellular sharp electrode recordings from CA3 pyramidal neurons were obtained with borosilicate glass microelectrodes (140–180 MΩ; WPI, Sarasota, US) filled with a potassium acetate solution (3 M) and connected to the headstage of an intracellular recording amplifier (Bio-logic VF180, Claix, France). Only data from neurons with both, stable resting membrane potential (RMP) with values ≤ −60 mV in the absence of direct current (DC) holding currents, and presenting overshooting action potentials, were collected for analysis. Spike amplitude, afterdepolarization and afterhyperpolarizing potentials were measured relative to threshold.

Excitatory and inhibitory postsynaptic potentials (EPSP and IPSP, respectively) were elicited orthodromically by stimulating the lateral fimbria where septal afferents to CA3 hippocampal neurons are mainly found (Alonso and Kohler, 1984; Amaral and Lavenex, 2007). For that purpose a monopolar stainless steel electrode (2 MΩ of effective resistance; WPI, Sarasota, US) and a programmable stimulator (MASTER-8, A8, A.M.P.I., Jerusalem, Israel) were used. Single, cathodal, square-wave pulses of 100–200 μs duration and 100–500 μA intensity were adjusted to subthreshold values for orthodromic spike generation. Postsynaptic potentials were characterized according to their amplitude (as a function of the RMP) and latency. Since horizontal slices were obtained at different level and angle, sometimes the location of the electrode along lateral fimbria had to be changed to evoke the characteristic triphasic response.

### Identification of stimulation and recording sites

Recorded neurons were identified following procedures described elsewhere (Navarro-Lopez et al., 2004). Briefly, selected neurons were stained by the intracellular injection of biocytin diluted in a 2 M potassium acetate solution, using positive current pulses of 0.2 nA for 6 min. Slices were fixed, and cut in sections (40 μm) using a freezing microtome (HM400R, Microm, Heidelberg, Germany). Sections were incubated with avidin-biotin-peroxidase complex (ABC, Vector Labs., Burlingame, US). 3,3′-Diaminobenzidine was used as chromogen for visualization of the biocytin complex. Sections were counterstained with cresyl violet. Neuron was reconstructed from serial sections using a graphic design software. Photographs were superimposed and orientated to obtain the best fit between the corresponding sectioned elements.

### Drugs

All chemicals used in this study were purchased from Sigma (Poole, UK) and Tocris (Biogen Científica, Spain) and applied by superfusion in the ACSF. The chemicals used were amyloid-β peptides (Aβ_25–35_ and the reverse Aβ_35–25_), 6-cyano-nitroquinoxaline-2,3-dione (CNQX; a potent, competitive AMPA-kainate receptor antagonist), 2-amino-5-phosphonovalerate (APV; a specific blocker of NMDA receptors), Bicuculline Methiodide (specific blocker of GABA_A_ receptors), (RS)-3-Amino-2-(4-chlorophenyl) propylphosphonic acid (Saclofen; blocker of GABA_B_ receptors), (RS)-4-Amino-3-(4-chlorophenyl) butanoic acid (Baclofen; agonist of GABA_B_ receptors), Tetrodotoxine (TTX; voltage dependent sodium channel blocker), Tertiapin-Q (selective blocker of GirK channels) and 2-methyl-2,4-pentanediol (MPD, agonist of GirK channels).

### Preparation of Aβ peptides solutions

Aβ_25–35_ and Aβ_35–25_ peptides were prepared as previously (Ashenafi et al., 2005; Santos-Torres et al., 2007). Briefly, the peptides were dissolved to 1 mM in bidistilled water and stored in aliquots at −20°C. Then aliquots were diluted in ACSF to required concentration and incubated for 24 h at 37°C before experiments were performed (Peña et al., 2010; Leao et al., 2012).

### Data storage and statistical analysis

Sharp electrode data were acquired online with the help of a CED 1401 interface (CED, Cambridge, UK), and stored on a personal computer (sample frequency 12.5 kHz). Analysis in both cases was performed using the MiniAnalysis Program, version 6.0.3 (Synaptosoft, Decatur, US). Unless otherwise indicated, the electrophysiological data are always expressed as mean ± standard error of the mean (SEM), and n represents the number of averaged neurons. Synaptic potentials were averaged (≥5) before quantitative analysis. Statistical analysis of collected data was performed using either Student's *t*-test or non-parametric test (Mann-Whitney *U*-test), accordingly with data distribution. When necessary, one-way ANOVA or equivalent non parametric test (Kruskal-Wallis test) and *post-hoc* analysis were performed. Statistical significance was determined at a level of *p* ≤ 0.05.

## Results

### Electrophysiological characterization of recorded neurons and their synaptic response to fimbria stimulation

**Figure 1:**
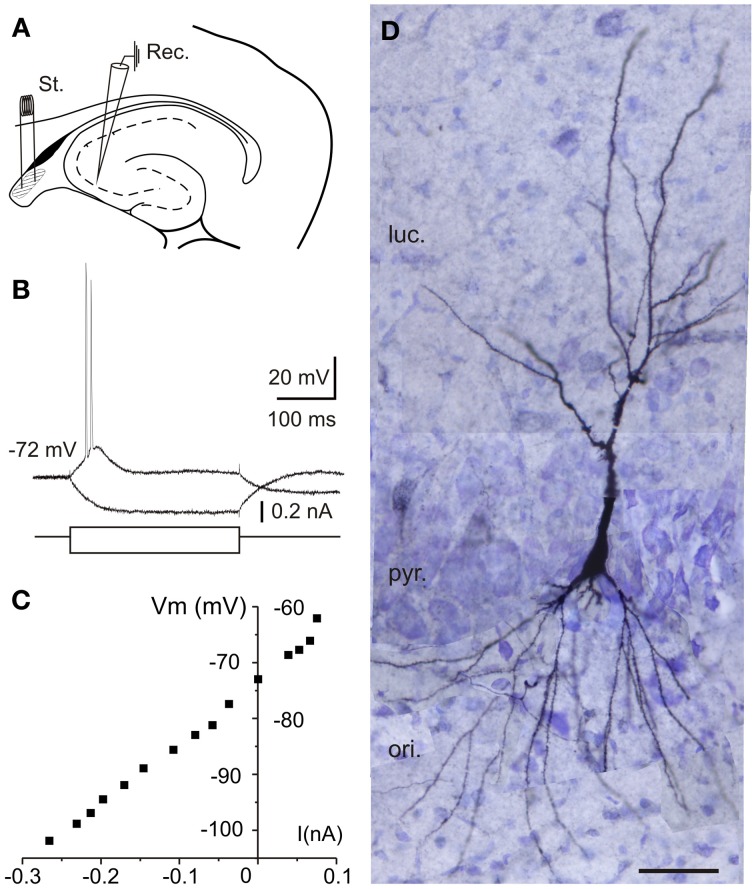
**Location and electrophysiological characterization of the recorded neurons in hippocampal slices. (A)** Experimental design. Diagram of stimulation and recording sites in an hippocampal horizontal section. Schematic location of recording (Rec.) and stimulating (St.) electrodes is shown. Stimuli were applied to the lateral part of the fimbria (shaded area). **(B)** Response of a CA3 neuron to depolarizing pulses consisted of two to five spikes with marked spike frequency adaptation with a depolarizing current pulse (100 pA, 300 ms) while hyperpolarizing current pulse injection (−160 pA, 300 ms) induced a hyperpolarizing response that allowed us monitoring the input resistance during the experiments. **(C)** Current-voltage (*I*-*V*) relationships for the pyramidal neuron recorded in **(B)**. **(D)** Reconstruction of the CA3 neuron recorded in **(B,C)** labeled with biocytin after intracellular recording from 40-μm-thick serial sections. Note the pyramidal morphology of the injected hippocampal cell. ori, *stratum oriens*; pyr, *stratum pyramidale*; luc, *stratum lucidum*; Scale bar 50 μm.

This study comprises 110 intracellular recordings from pyramidal CA3 neurons (Figure 1), selected on the basis of their RMP (≤ −60 mV) and monosynaptic activation from the fimbria. Recorded neurons did not exhibit action potentials spontaneously at RMP values (−72.5 ± 1.8 mV). The input resistance (Ri) of the neurons was 113.4 ± 6.7 MΩ and the membrane time constant was 77.2 ± 25.4 ms. The direct activation of these neurons by depolarizing current injections (0.1–0.6 nA ; 300 ms) evoked a series of two to five spikes with marked spike frequency adaptation and decreased amplitude and longer duration of the second spike relative to the first one (Figure 1B). The spike amplitude was 101.1 ± 3.2 mV. These characteristics, together with neuronal morphology (Figure 1D) and other electrophysiological properties such as the presence of triphasic afterhyperpolarization (fAHP: 5.6 ± 0.8 mV; mAHP: 10.5 ± 1.5 mV; sAHP: 17.6 ± 1.3 mV) or afterdepolarization (ADP: 3.6 ± 0.5 mV), characterize the principal pyramidal-like neurons widely described in the hippocampus (Spruston and Johnston, 1992; Wittner et al., 2007). The location of selected neurons (*n* = 10) filled with biocytin is illustrated in Figure 1A. The morphology corresponds to pyramidal neurons in CA3 region of the hippocampus. The cell body is located into the *stratum pyramidale* and the visible basal dendrites on *stratum oriens* (Figure 1D).

**Figure 2:**
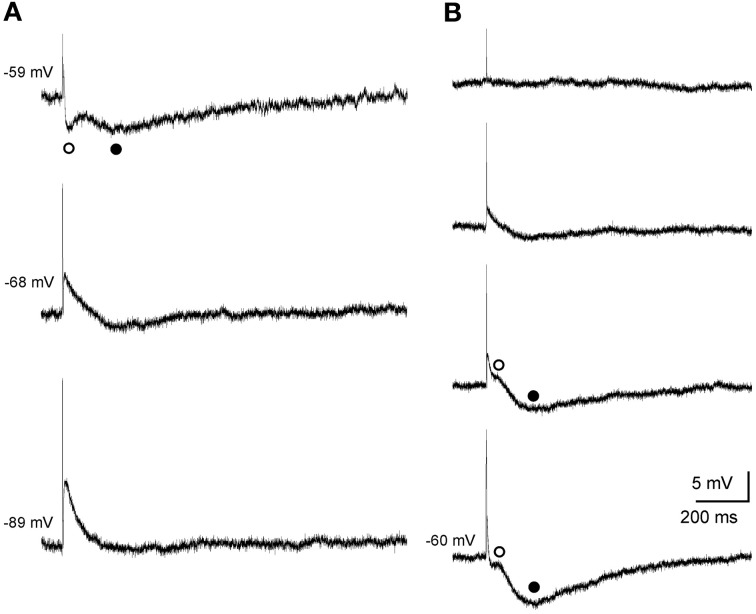
**Postsynaptic septohippocampal response in CA3 pyramidal neurons. (A)** Effect of membrane potential variations on the amplitude of the early and late IPSPs evoked by orthodromic activation in a pyramidal CA3 neuron. Stimulation of the fimbria elicited an EPSP followed by an early (open circles) and late (closed circles) IPSP. Traces shown are the average of five responses. **(B)** Effect of varying the intensity of fimbria stimulation on the complex postsynaptic response recorded in another neuron at a membrane potential of −60 mV. From top to bottom, traces represent synaptic responses which were evoked by progressive increments in fimbria stimulation. Stimulation of the fimbria at a low intensity evoked only EPSP followed by an early (◦, open circles) IPSP. Delivery of stimulation at higher intensities resulted in the elicitation of a subsequent late (•, closed circles) IPSP. The approximate reversal potential for both inhibitory components was early −61.1 mV and late −80.0 mV.

Single subthreshold stimulation of the fimbria evoked stereotyped triphasic synaptic responses in CA3 pyramidal cells (Figure 2). The initial response was a fast EPSP, which occurred at a latency of 6.5 ± 0.6 ms following stimulus offset, suggesting the monosynaptic nature of the connection. The size of the EPSP was graded with the stimulus intensity and it increased in amplitude when elicited at progressively more negative membrane potentials (Figure 2A). The EPSP was followed by a rapidly developing hyperpolarization (early IPSP, Figures 2A,B) that reached its peak amplitude 30.4 ± 1.4 ms (*n* = 11) following fimbria stimulation. Finally a second hyperpolarization (late IPSP, Figures 2A,B) following the early IPSP, had a latency to peak amplitude of 247.4 ± 6.6 ms (*n* = 44). The amplitudes of both, IPSPs and EPSP, varied with membrane potential (Figures 2A,B), allowing us to determine the approximate reversal potential for both inhibitory components (early −61.1 mV and late −80.0 mV).

**Figure 3:**
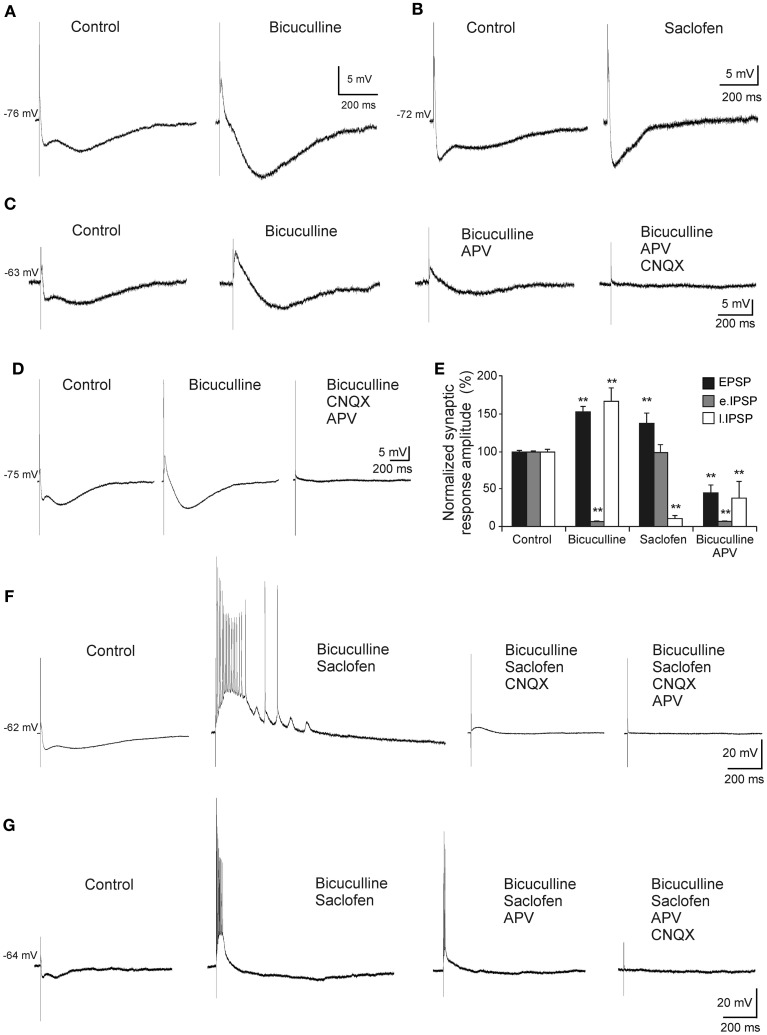
**Pharmacological characterization of septohippocampal synaptic response. (A)** Recordings from a pyramidal CA3 neuron illustrating a marked reduction of the early IPSP after perfusion with bicuculline (10 μM; specific blocker of GABA_A_ receptors). **(B)** Blocking of the late IPSP after perfusion with saclofen (200 μM; blocker of GABA_B_ receptor) was accompanied by a mild increase of the early IPSP and EPSP. **(C)** The increase in the amplitude of EPSP and late IPSP produced by perfusion of bicuculline was reduced by APV application (50 μM; specific blocker of NMDA receptor). Membrane potential was maintained at values more positives than resting membrane potential to assure NMDA receptors functionality. Synaptic response was completely removed with the addition of CNQX (10 μM; competitive non-NMDA receptor antagonist). **(D)** Blockade of the early IPSP with bicuculline (*n* = 4) was associated with a marked increase in amplitude of EPSP and late IPSP. Excitatory and inhibitory responses were blocked by CNQX and APV. The cells were held at −75 mV (a membrane potential where NMDA receptor-mediated currents are null). **(E)** Histograms with relative mean amplitude as percentage of control of the different components of the complex synaptic response (EPSP; early, e.IPSP; and late, l.IPSP) under pharmacological conditions described in **(A–D)** (^**^p < 0.001). **(F)** Both, early and late IPSPs were blocked by bicuculline and saclofen, respectively, while a large repetitive burst of action potential appeared (*n* = 4). At membrane potential values that assured NMDA activation, this epileptic-like activity could be removed by CNQX. Finally, residual excitatory NMDA response was eliminated by APV perfusion. **(G)** During the epileptic-like activity induced by both inhibitory components elimination, and at membrane potential values that led NMDA receptor activation, APV perfusion reduced the size of the epileptic response that had to be removed by addition of CNQX (*n* = 4).

In order to determine the nature of the complex response, a pharmacological dissection of the postsynaptic potential components was performed (Figure 3). The early IPSP was blocked by bicuculline (10 μM, *n* = 6), a specific blocker of GABA_A_ receptors (Figures 3A,C–E) whereas late IPSP was removed by saclofen (200 μM; *n* = 5), a specific blocker of GABA_B_ receptors (Figures 3B,E). The excitatory component was increased by early IPSP block with bicuculline (*n* = 9; Figures 3A,C–E) and presented a glutamatergic nature acting mainly on non-NMDA (*n* = 5; Figure 3D) receptors, since although its complete elimination required CNQX (10 μM) and APV (50 μM) the cells were held at −75 mV (a membrane potential where NMDA receptor-mediated currents are null). However, when recorded cells were held at more positive values than RMP (*n* = 4; Figure 3C), NMDA channels were entirely functional and perfusion with APV decreased both bicuculline-enhanced responses, EPSP and late IPSP (Figure 3E), suggesting the participation of NMDA receptors in the response. As previously, complete blockage of EPSP also required CNQX (Figure 3C). In this regard, the pharmacological elimination of glutamatergic responses with CNQX plus APV also abolished the inhibitory response (Figures 3C,D) suggesting that IPSPs were produced by interneurons activation.

On the other hand, when both inhibitory components were removed an epileptiform-like discharge was generated (Figures 3F,G). This response could reach a firing frequency of 90 Hz and showed a glutamatergic nature acting mainly on non-NMDA receptors, since CNQX completely abolished it (Figure 3F) whereas APV only could block it partially (Figure 3G).

These results indicate that the triphasic complex response involves excitatory and inhibitory neurotransmission mediated by glutamate and GABA receptors activation, suggesting that a precise tuning is required for information processing at this synapse.

### Aβ_25–35_ differential effects on membrane properties

**Figure 4:**
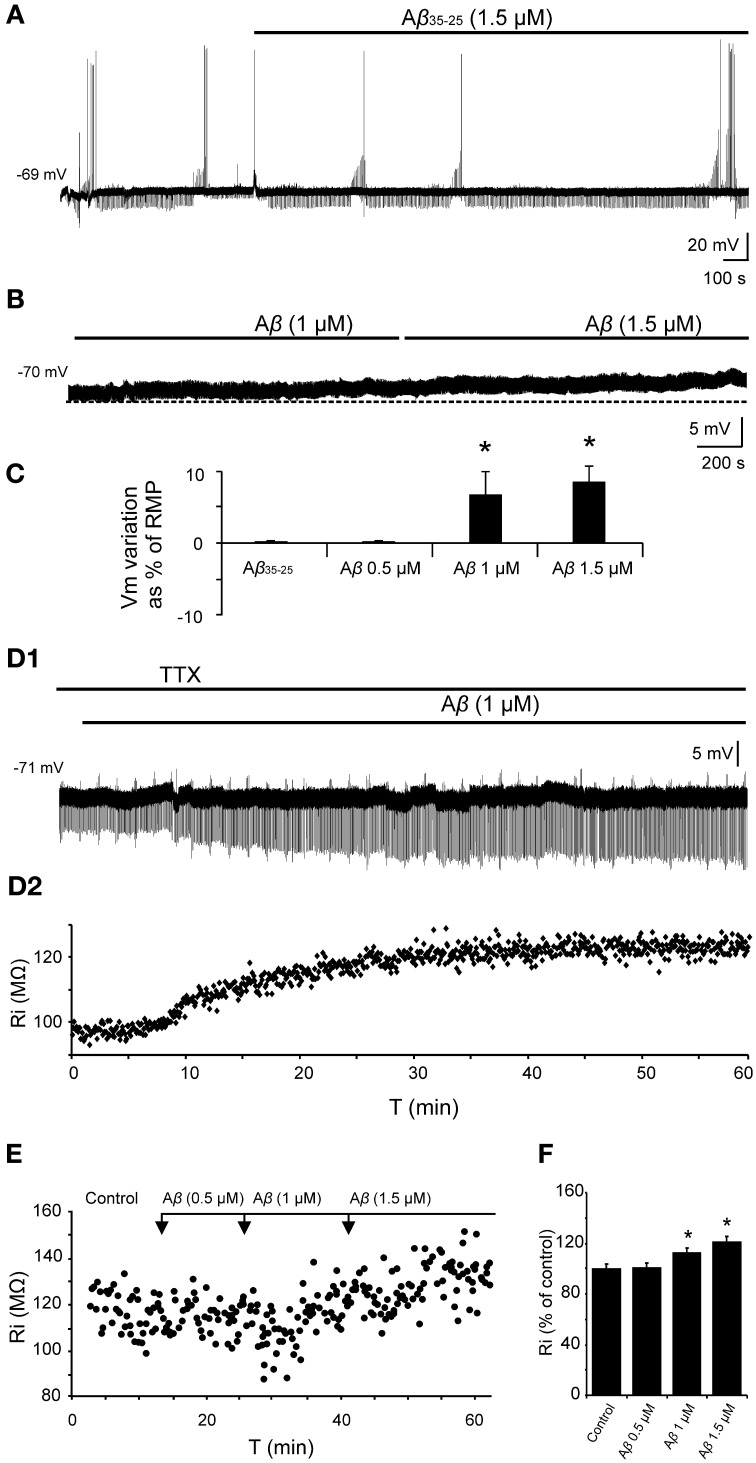
**Effects of Aβ_25–35_ on membrane potential and Ri of CA3 neurons. (A)** Recording of a CA3 pyramidal neuron during the perfusion of the reverse sequence of the peptide, Aβ_35–25_, used as negative control (*n* = 4). **(B)** Depolarization of membrane potential induced by perfusion of Aβ in a recorded pyramidal CA3 neuron (*n* = 10). **(C)** Plot of the membrane potential variations as a percentage of resting membrane potential (RMP). Perfusion of Aβ_35–25_ 1 μM or Aβ_25–35_ 0.5 μM did not induce any significant change in the membrane potential or Ri. Aβ_25–35_ higher concentrations induced the cells to depolarize (1 μM; 6.8 ± 3.2 %, *n* = 4; 1.5 μM, 8.6 ± 2.2 %, *n* = 10). **(D1)** Time course of Aβ effects on Ri in a CA3 pyramidal neuron after perfusion with TTX. The membrane potential was held at its RMP value by direct current (DC) holding current injection to cancel out the depolarization. **(D2)** For the same neuron, each point represents the Ri during the recording in **(D1)**. Membrane potential was held at −71 mV. **(E)** Plot showing the time course of the effects of Aβ_25–35_ concentration increase on the Ri of a CA3 pyramidal neuron. Note that recordings last for a very long time. **(F)** Histogram with mean values in percentage for Ri (*n* = 10) at different Aβ_25–35_ concentration (^*^p < 0.05).

In all cases, the recordings were stabilized for at least 10 min. During this time, characterization of firing pattern, membrane potential, Ri and synaptic responses were performed. The specificity of the Aβ_25–35_ peptide action was confirmed by the use, as negative control, of the reverse sequence Aβ_35–25_ (1.5 μM), without any noticeable effect (Figures 4A,C). Then, slices were perfused with increasing concentrations of Aβ_25–35_ (0.5, 1.0, and 1.5 μM) for at least another 10 min at each concentration.

No significant differences were found in spike amplitude [*F*_(3, 50)_ = 2.62, *p* = 0.062], threshold [*F*_(3, 50)_ = 2.14, *p* = 0.108], ADP [*F*_(3, 22)_ = 0.576, *p* = 0.638], fAHP [*F*_(3, 31)_ = 1.22, *p* = 0.320], or sAHP [*F*_(3, 33)_ = 1.625, *p* = 0.204] after perfusion with Aβ_25–35_ at increasing concentrations. However, as shown in Figures 4B,C, a significant depolarization was observed when Aβ_25–35_ was applied (1 μM; 4.3 ± 2.3 mV; *n* = 4; and 1.5 μM; 6.2 ± 1.6 mV; *n* = 10). The membrane potential of the recorded neurons was maintained at its RMP value by DC holding current injection to cancel out the depolarization induced by Aβ_25–35_ (Figure 4D). These variations in current injection were statistically significant at 1.0 μM (*t* = 2.557, *p* = 0.021) and 1.5 μM (*t* = 4.301, *p* < 0.001) concentrations and no difference was observed at 0.5 μM concentrations (*t* = 0.842, *p* = 0.412). Additionally, Aβ_25–35_ also produced a significant increase in the relative Ri (% = Ri recorded/Ri control ^*^100; *n* = 16) (Figures 4D–F) at 1.0 μM concentration (*t* = −2.635, *p* = 0.018) and 1.5 μM (*t* = −3.236, *p* = 0.007) whereas no differences were found at 0.5 μM (Mann–Whitney U Statistic = 10.000, *p* = 0.690) (Figures 4E,F).

To determine the synaptic location of these Aβ_25–35_ effects, slices were perfused with TTX and any afferent synaptic activity was blocked. In these conditions, superfusion of the slice with Aβ_25–35_ was able to evoke both, depolarization and Ri increasing (Figure 4D) of intracellularly recorded CA3 pyramidal neurons, suggesting a postsynaptic location for the Aβ_25–35_ action mechanism (*n* = 5).

### Differential effects of Aβ_25–35_ on fimbria-CA3 synaptic response

**Figure 5:**
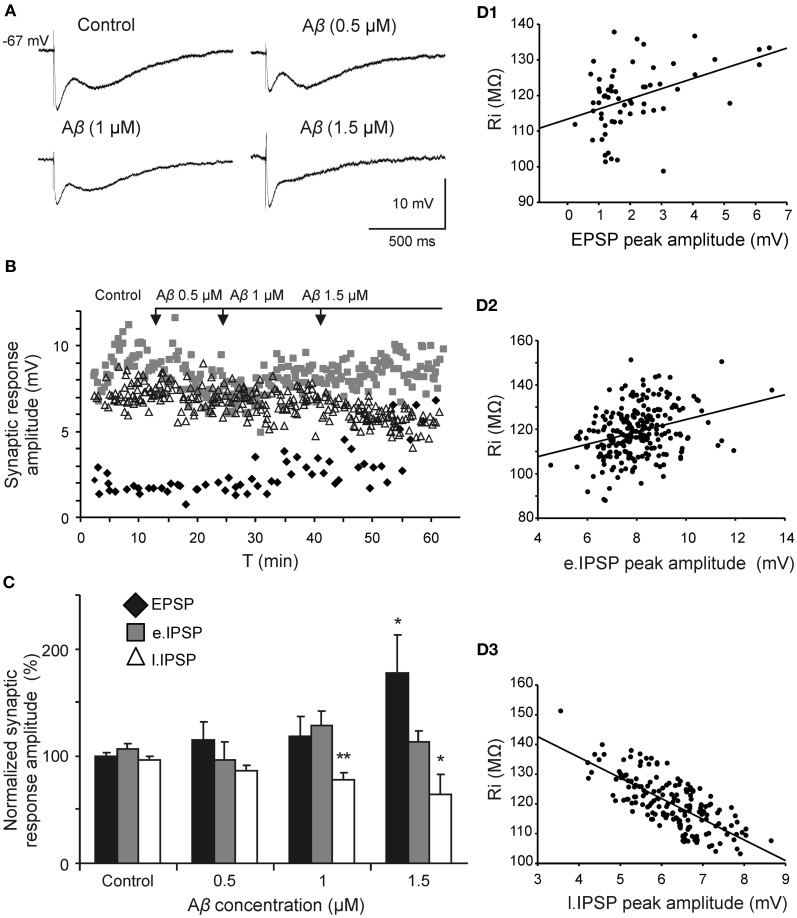
**Selective effect of Aβ_25–35_ on different components of the complex postsynaptic response recorded in CA3 pyramidal neurons by fimbria stimulation. (A)** Evoked responses obtained in a pyramidal CA3 neuron by fimbria orthodromic stimulation (control) and during perfusion of different concentrations of Aβ_25–35_ (0.5, 1, and 1.5 μM). The reduction of the late IPSP after perfusion with Aβ_25–35_ has been shown to be concentration-dependent. **(B)** Plot displaying the time course of Aβ_25–35_ perfusion effects on the amplitude (in mV) of the different components of the complex response (EPSP, black diamonds; early IPSP, gray squares; late IPSP, white triangles; see color code in **C**). **(C)** Histograms with relative mean amplitude (*n* = 20) of the different components of the complex synaptic response (EPSP; early, e.IPSP; and late, l.IPSP) 10 min after Aβ_25–35_ perfusion (0.5, 1, and 1.5 μM). Significant differences were found for EPSP at 1.5 μM Aβ_25–35_ and for late IPSP at 1–1.5 μM Aβ_25–35_. **(D)** Correlation analysis of Aβ_25–35_ perfusion on different components of the complex postsynaptic response vs. Ri values. Data showed a higher correlation between Aβ-induced Ri increase and late IPSP amplitude (*R* = −0.73, *p* < 0.001; **D3**) than Aβ-induced Ri increase and EPSP (*R* = 0.42, *p* < 0.001; **D1**) or early IPSP (*R* = 0.31, *p* < 0.01; **D2**)(^*^p < 0.05; ^**^p < 0.01).

Since Aβ has widely shown to exert its effects through septohippocampal network impairing, we examined whether this peptide altered the fimbria-CA3 complex postsynaptic response (*n* = 20). In the experiment shown in Figure 5A, superfusion of Aβ_25–35_ produced a significant decrease of the late IPSP component (1 μM; *t* = 2.532, *p* = 0.030 and 1.5 μM; *t* = 2.519, *p* = 0.036) that was neither observed at 0.5 μM (*t* = 1.133, *p* = 0.295) nor on early IPSP (*H* = 2.578; *p* = 0.461). However, the late IPSP reduction was associated with an increase in the excitatory response observed at concentration of 1.5 μM (*t* = −2.503, *p* = 0.046) but not at lower concentrations (Figures 5B,C). These results indicate a possible mechanism to imbalance the particular excitatory/inhibitory tuning in the septohippocampal system, and therefore a differential Aβ_25–35_ effect, according to the specific neurotransmission system involved.

Due to this differential Aβ_25–35_ effect on both inhibitory components, the late component reduction may not be attributable to a decreased neurotransmitter release since GABA_A_ component amplitude was maintained. This result pointed out to a selective postsynaptic action on GABA_B_ complex.

### Aβ_25–35_ effects can be explained by a reduction in the conductance of GirK channels coupled to GABA_B_ receptor

**Figure 6:**
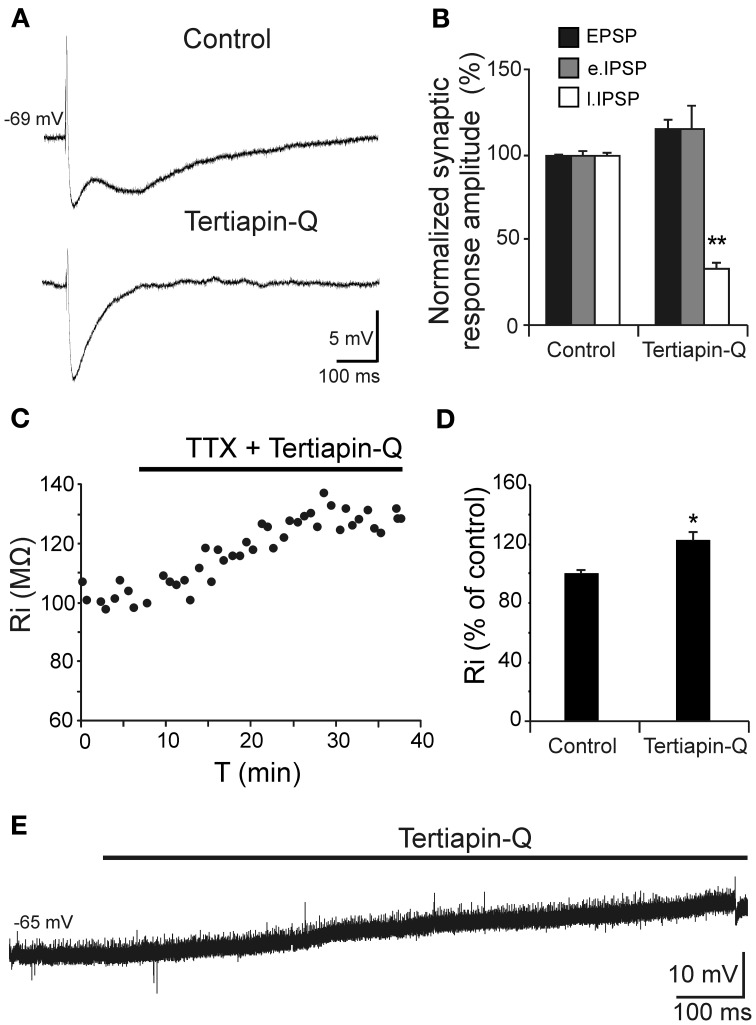
**Effects of the selective GirK channel antagonist tertiapin-Q on CA3 pyramidal neurons complex response to fimbria stimulation. (A)** Selective blockade by tertiapin-Q of the late IPSP recorded in CA3 pyramidal neurons after fimbria stimulation. Superfusion of the GirK antagonist tertiapin-Q (0.5 μM; selective blocker of GirK channels) selectively blocked the late IPSP but did not reduce the early IPSP that was elicited by fimbria stimulation. **(B)** Histograms with relative mean amplitude (*n* = 5) of the different components of the complex synaptic response (EPSP; early, e.IPSP; and late, l.IPSP) 40 min after tertiapin-Q perfusion. Significant differences were found for late IPSP. **(C)** Results of an experiment in another neuron designed to assess the postsynaptic effects of tertiapin-Q on Ri of CA3 pyramidal neurons. Perfusion of TTX (*n* = 5; 1 μM; voltage-dependent sodium channel blocker) blocked afferent neurotransmission and therefore any effect of tertiapin-Q took place at postsynaptic location. Note the significant Ri increase after 30 min. **(D)** Histogram with mean values in percentage for Ri (*n* = 6) after tertiapin-Q perfusion during 40 min (^*^p < 0.05). **(E)** Effect of tertiapin-Q on CA3 pyramidal neurons membrane potential (*n* = 5). Chart record shows that superfusion of tertiapin-Q (0.5 μM) produced a marked depolarization when applied at resting membrane potential (−65 mV). ^**^p <0.01.

Depolarization caused by Aβ_25–35_ was associated to Ri increase and therefore, linked to a possible decrease in membrane conductance, which may very likely involve in ion channels closing. Given the correlation between reduction in GABA_B_ component and Ri increase shown in Figure 5D, we investigated whether these Aβ_25–35_ effects could be mediated by conductance reduction of GABA_B_ effector, GirK channel. We found that GirK blockade by its selective antagonist, tertiapin-Q (0.5 μM), not only removed the GABA_B_ component (late IPSP; Figures 6A,B) of the synaptic response, but also induced a significant increase in Ri (Figures 6C,D; 122.5 ± 5.4%; *t* = −3.264; *p* = 0.022) as well as membrane depolarization (Figure 6E; 9.1 ± 2.6 mV; *n* = 5; *t* = 8.69, *p* < 0.001), therefore mimicking all Aβ_25–35_ effects previously described.

**Figure 7:**
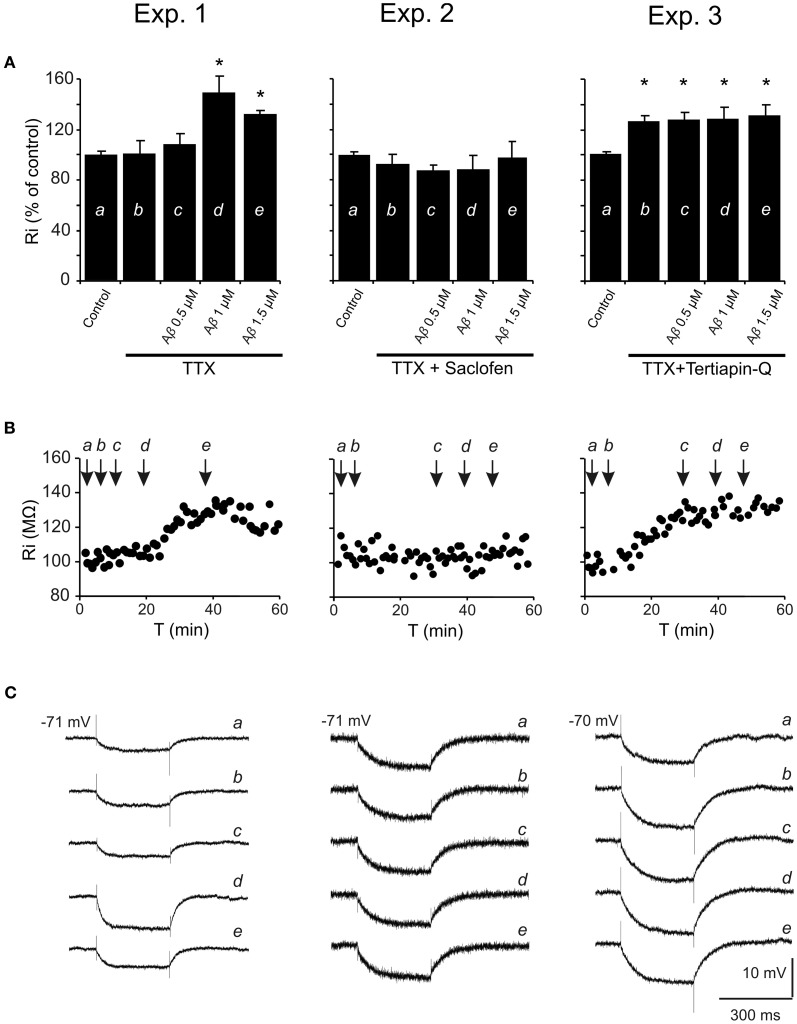
**Histograms with Ri mean values in CA3 pyramidal neurons for increasing Aβ_25–35_ concentrations. (A)** Normalized mean value of Ri as % of control after perfusion with three different Aβ_25–35_ concentrations in three different experimental conditions (Exp. 1–3). In all cases, TTX pre-treatment (1 μM; voltage-dependent sodium channel blocker) was unable to prevent Aβ_25–35_ effects on Ri, suggesting a postsynaptic location for Aβ_25–35_ action. Exp. 1: Significant increase on Ri was recorded after 1 and 1.5 μM Aβ_25–35_ perfusion (*n* = 16), even in the presence of TTX. Exp. 2: After perfusion of both TTX and saclofen (to block postsynaptic GABA_B_ receptors), Aβ_25–35_ was unable to evoke any significant modification in Ri (*n* = 6). Exp. 3: As in the experiment showed in Figure 6B, TTX perfusion together with the selective antagonist of GirK channels, tertiapin-Q (0.5 μM), induced a significant increase in Ri compared to control values. However, when tertiapin-Q was perfused and GirK channels blocked, Aβ_25–35_ became unable to induce any additional Ri increase (*n* = 6). Control value for Ri was normalized to 100%. Data show mean ± SEM. ^*^p < 0.05. Lower-case letters (*a–e*) indicate the five pharmacological conditions during each experimental treatment. **(B)** Representative examples for time course of Ri recorded in experimental conditions (Exp. 1–3). The arrows indicate the time point at which drugs were applied during the recordings. **(C)** Representative examples of recordings were expanded in time to show the changes in membrane potential during the presentation of hyperpolarizing pulses. This protocol allowed us to monitor Ri during the whole recording and to study the effect of different pharmacological treatments represented by lower-case letters (*a–e*).

To determine the mechanism involved in the postsynaptic reduction of the late IPSP amplitude and Ri increase, pharmacological blockage of different receptors/channels was performed and effects of increasing Aβ_25–35_ concentrations on Ri were evaluated (Figure 7). The blockade of synaptic transmission by TTX did not produce significant changes in Ri (Mann-Whitney U, *p* = 0.700) compared to control (Figures 7A–C, Exp. 1–3). Then, Aβ_25–35_ perfusion produced a significant increase in Ri (*n* = 4) at high concentrations (Figure 7, Exp. 1; 1.0 μM, *t* = −7.424; *p* = 0.018 and 1.5 μM, *t* = −7.519; *p* = 0.002), which was not observed at 0.5 μM (Mann-Whitney U, *p* = 1.00). These results indicate a postsynaptic Aβ_25–35_ mechanism that quite likely involves a reduction in ion channels conductance.

On the other hand, since saclofen perfusion prevented Aβ_25–35_-induced changes on Ri (*n* = 6; *H* = 3.964, *p* = 0.411; Figure 7, Exp. 2), an interaction with GABA_B_ receptors might be assumed. However, although saclofen also induced a reduction on late IPSP amplitude (see Figures 3B,E), in the presence of TTX it did not exhibit any noticeable effect on membrane potential (1.7 ± 2.8 mV; *t* = −0.541; *p* = 0.617; non illustrated) or Ri (Figure 7, Exp. 2; 94.5 ± 7.3%; *t* = 1.198; *p* = 0.285), in contrast to Aβ_25–35_. Altogether, these results suggest that Aβ_25–35_ exerts its effects acting preferentially on postsynaptic GirK channels, instead of GABA_B_ receptors.

In accordance with this hypothesis, Aβ_25–35_ was found to be unable to generate additional increase on Ri after postsynaptic blockage of GirK channels by tertiapin-Q [Figure 7, Exp. 3; *n* = 6; *F*_(3, 21)_ = 0.129, *p* = 0.941], suggesting that Aβ-induced Ri increase may be associated to a reduction in GirK channel conductance. However, because conductance depends, among others, on the number of channels, their open probability or membrane voltage, the methodology used in the present study has some limitations to determine the exact mechanism for Aβ-mediated Ri increase. In addition, in some experiments, it was necessary to inject DC to maintain a stable membrane potential and to prevent depolarization. Previous studies had reported that depolarization mechanisms induced by Aβ might involve activation of glutamatergic receptors (Blanchard et al., 2002a,b). In order to control these variables, tertiapin-Q was perfused together with glutamatergic antagonists CNQX (10 μM) and APV (50 μM). This protocol not only prevented the Aβ-mediated increase in Ri, but also abolished the dependence of DC injection to compensate membrane depolarization (*n* = 4; H = 3.709, *p* = 0.447; non illustrated). In contrast, perfusion with CNQX, APV and the GABA_A_ blocker, bicuculline (10 μM), was not able to prevent the Ri increase induced by Aβ_25–35_ (*n* = 4; H = 21.681; *p* < 0.001; non illustrated) further suggesting an effect of Aβ_25–35_ on GirK channels.

### Effects of Aβ_25–35_ on the hyperpolarization mediated by GABA_B_-GirK activation

**Figure 8:**
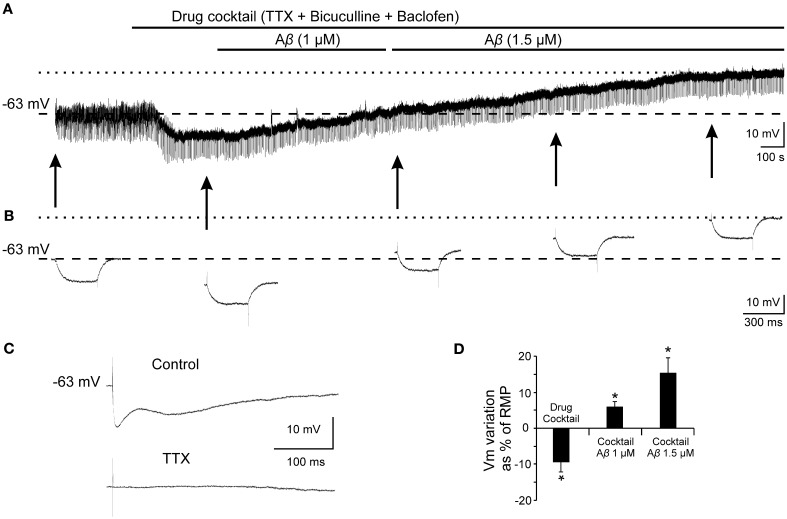
**Effects of Aβ_25–35_ on postsynaptic hyperpolarization induced by activation of GABA_B_ receptors. (A)** Membrane potential recording from a CA3 neuron (top) after perfusion with TTX (1 μM; voltage-dependent sodium channels blocker), bicuculline (10 μM; specific blocker of GABA_A_ receptor) and baclofen (15 μM; agonist of GABA_B_ receptor). This treatment (*n* = 4) produced a membrane hyperpolarization depending on GABA_B_ postsynaptic receptors activation. Perfusion with Aβ_25–35_ (1 and 1.5 μM) markedly induced the membrane to depolarize. **(B)** Intracellular hyperpolarizing current pulses. The arrows indicate the time points at which recordings were expanded in time to show the changes in the membrane potential during the presentation of hyperpolarizing pulses. This protocol allowed us to monitor Ri during the whole recording and check the viability of the neurons. Dashed lines in **(A)** and **(B)** indicate membrane resting potential. Maximum membrane potential evoked by Aβ_25–35_ superfusion is indicated by dotted lines. **(C)** In the same neuron, fimbria stimulation elicited the characteristic triphasic postsynaptic response before drugs cocktail perfusion. This complex postsynaptic potential was completely removed by TTX (1 μM; voltage-dependent sodium channels blocker) perfusion. **(D)** Plot of the membrane potential variations as a percentage of resting membrane potential (RMP) under pharmacological conditions presented in **(A)**. TTX, bicuculline and baclofen (drug cocktail) induced pyramidal neurons to hyperpolarize while Aβ_25–35_ produced a noticeable depolarization (*n* = 4; 21.4 ± 5.9 mV). ^*^p < 0.05.

Given that, Ri changes may depend on multiple factors and might be affected by Aβ acting on different ion channels and receptors, we designed a protocol to specifically evaluate the effects of Aβ_25–35_ on GABA_B_ response. To verify whether Aβ_25–35_ affects the postsynaptic response mediated by GABA_B_ receptor activation, we used a drug cocktail including: TTX to block synaptic transmission, bicuculline to block GABA_A_ receptors activation and baclofen to stimulate GABA_B_ receptors (Figure 8). Cocktail application in the slice produced a postsynaptic membrane hyperpolarization in recorded CA3 pyramidal neurons (Figures 8A,D; −9.5 ± 2.8% of the RMP value). When membrane potential was stabilized, Aβ_25–35_ perfusion induced a pronounced depolarization (Figures 8A,D; 15.5 ± 4.3 % of the RMP value; *n* = 4), which confirms that Aβ_25–35_ reduces the postsynaptic GABA_B_ response in a concentration and time dependent manner. But Aβ_25–35_ action on this GABA_B_ response might also be explained by an effect on its final effector, GirK, which would also underlie the already described Aβ_25–35_ effects on Ri and membrane depolarization.

**Figure 9:**
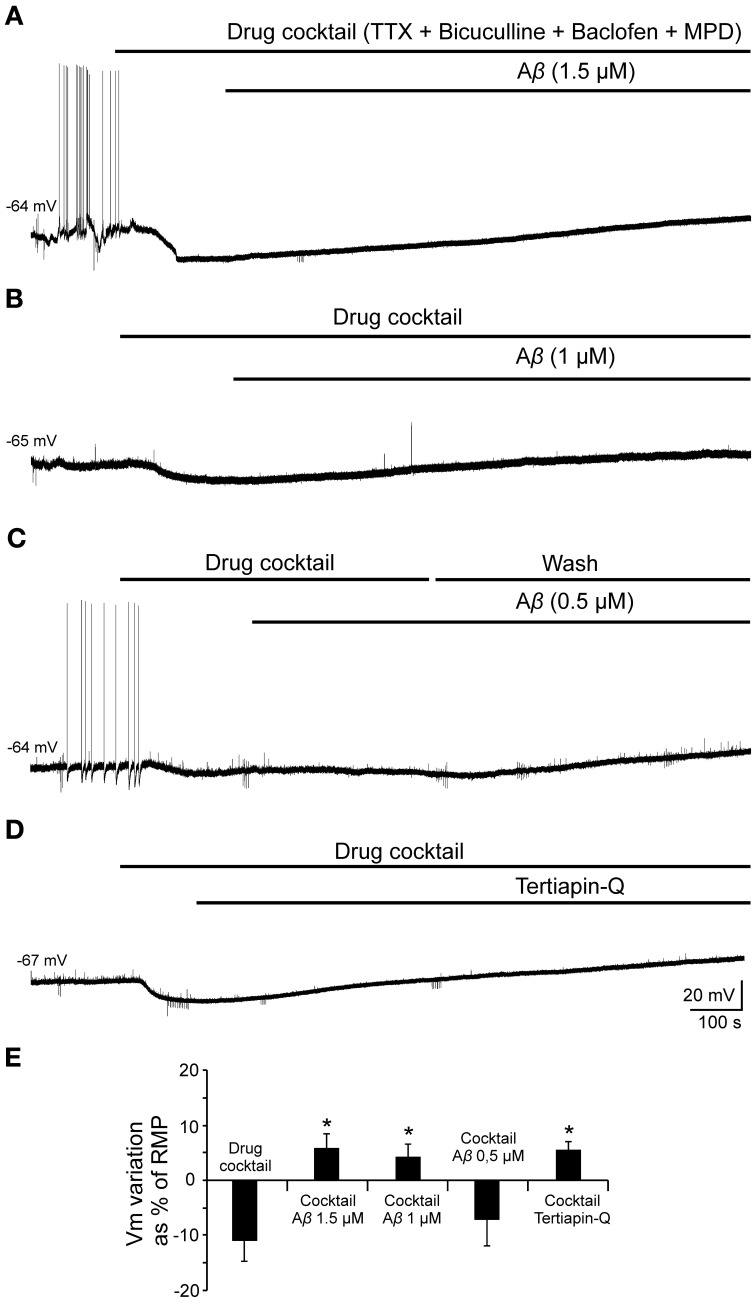
**Effects of Aβ_25–35_ perfusion on the response evoked by pharmacological co-activation of GirK channels and GABA_B_ receptors. (A,B)** GirK/GABA_B_ postsynaptic hyperpolarization was induced (*n* = 15) by perfusion of drugs cocktail including TTX (1 μM; voltage-dependent sodium channels blocker), bicuculline (10 μM; specific blocker of GABA_A_ receptors), baclofen (15 μM; agonist of GABA_B_ receptors), and MPD (50 mM; GirK channel agonist) perfusion. Aβ_25–35_ 1.5 μM (*n* = 3) and 1.0 μM (*n* = 4)(**A** and **B**, respectively) evoked a postsynaptic depolarization. **(C)** Using the same protocol, Aβ_25–35_ 0.5 μM was not able to produce this depolarization. The cocktail hyperpolarizing effect on membrane potential disappeared after cell washing with bathing solution (*n* = 4). **(D)** Tertiapin-Q (*n* = 4; 0.5 μM; selective blocker of GirK channels), the specific antagonist of GirK channels, induced a depolarization of the postsynaptic hyperpolarization mediated by GirK/GABA_B_ activation, reproducing Aβ_25–35_ effects. **(E)** Plot showing the membrane potential changes as a percentage of resting membrane potential (RMP) under pharmacological conditions presented in **(A–D)**. ^*^p < 0.05.

In order to validate this hypothesis and evaluate the effect of Aβ_25–35_ on GirK response, we used a GirK channel agonist, MPD (Aryal et al., 2009). Bath application of the previous drugs cocktail together with MPD (50 mM) induced the cell to hyperpolarize by two postsynaptic mechanisms, GABA_B_ receptor activation and direct increase in GirK conductance (Figure 9; −11.2 ± 3.8% of the RMP value; *n* = 15). Then, perfusion of Aβ_25–35_ (1–1.5 μM) removed the hyperpolarization mediated by GABA_B_-GirK stimulation (Figures 9A,E; 5.9 ± 2.7%; *n* = 3, Figures 9B,E; 4.2 ± 2.4%, *n* = 4), while this effect was not evident at 0.5 μM (Figures 9C,E; −7.2 ± 4.8% *n* = 4). In fact, the hyperpolarization could be eliminated when the cocktail was washed (Figure 9C). Finally, Aβ-induced depolarization was mimicked by tertiapin-Q, the specific antagonist of GirK channels (Figures 9D,E; 5.5 ± 1.5%; *n* = 4), indicating that Aβ_25–35_ directly affects GirK channels conductance.

## Discussion

Despite the importance that rhythms as an emergent property of neural network seem to have, few studies have investigated how Aβ induces injury and how it may contribute to impair septohippocampal oscillatory activity, which in turn may underlie the early symptoms typically observed in AD patients (Colom, 2006; Palop and Mucke, 2010; Villette et al., 2010; Rubio et al., 2012; Verret et al., 2012). The present study identifies alterations in GirK channels conductance of fimbria-CA3 synapse as a putative mechanism of Aβ-induced synaptic dysfunction observed in the septohippocampal system activity.

### Septohippocampal system and Aβ_25–35_ neurotoxicity

Previously, it has been proposed that Aβ_25–35_ constitutes the biologically active fragment of Aβ (Millucci et al., 2010), and has been shown to induce major neuropathological signs related to early stages of AD in rats (Klementiev et al., 2007). In addition, Aβ_25–35_ is reported to be more soluble and presents toxic effects more rapidly than the parent peptide Aβ_1–42_ (Varadarajan et al., 2001), and has widely been used as a very useful tool to explore acutely the pathophysiological events related with neuronal dysfunction induced by soluble Aβ forms (Ashenafi et al., 2005; Santos-Torres et al., 2007; Peña et al., 2010; Leao et al., 2012). But the most important advantage for present work is that Aβ_25–35_ does not form ion-permeable pores in neuronal membrane (Jang et al., 2010; Chang et al., 2011; Leao et al., 2012) which could alter our protocols, especially input resistance measurements.

From neuroanatomical and electrophysiological points of view, hippocampus receives different projections from the medial septum diagonal broca band mainly through lateral fimbria (Wyss et al., 1980; Alonso and Kohler, 1984; Colom, 2006; Amaral and Lavenex, 2007). Cholinergic neurons innervate pyramidal neurons and interneurons (Widmer et al., 2006), GABAergic fibers project onto interneurons (Freund and Antal, 1988; Chamberland et al., 2010) and glutamatergic projections contact with CA3 pyramidal cells (Huh et al., 2010). These septo-hippocampal circuits are involved in both, generating hippocampal *theta* rhythm, as well as in learning and memory processes. To maintain these high-level functions, a precise septohippocampal network activity requires of finely regulated excitatory and inhibitory neurotransmission (Buzsaki, 2002; Sotty et al., 2003; Borhegyi et al., 2004; Colom, 2006) whose alteration could lead to impairments that would be consistent with the deficits described for AD initial stages (Bland and Colom, 1993; Palop and Mucke, 2010). In this sense, it has been reported that in hippocampus, Aβ mainly induces aberrant inhibitory septohippocampal network activity (Palop et al., 2007; Villette et al., 2010, 2012). Therefore, although an Aβ effect on inhibitory neurotransmission might be expected, the putative mechanism to detune the network coordination of this system remains unclear.

Aβ_25–35_ was found not to affect the active properties of CA3 pyramidal recorded neurons suggesting that Aβ_25–35_ does not modify the conductances that mediate active membrane properties such as sodium or potassium voltage-gated channels for spike amplitude (Hille, 2001), BK/SK channels for AHPs (Sah and Faber, 2002) or R-type calcium channels for ADP (Metz et al., 2005). Similar results have also been shown in other AD related regions as amygdala (Ashenafi et al., 2005), septum (Santos-Torres et al., 2007), or cortex (Wang et al., 2009).

On the other hand, Aβ_25–35_ induced an Ri increase associated with membrane depolarization. We have previously showed that Aβ_25–35_ exerts variable effects on membrane potential and Ri in amygdalar pyramidal neurons, possibly due to a presynaptic mechanism (Ashenafi et al., 2005) and in septal neurons, depending on pre- and postsynaptic actions (Santos-Torres et al., 2007). In the present study, TTX was not able to prevent Aβ_25–35_ effects suggesting a direct effect of Aβ_25–35_ on CA3 pyramidal neurons membrane.

Previous research has shown that neurotransmission in the septohippocampal system is affected by Aβ through altering the *theta* oscillatory activity (Colom et al., 2010; Rubio et al., 2012), but studies at the synaptic level on the mechanisms underlying this alteration have not been exhaustively performed. Thus, the possibility of studying in a single preparation and in a particular synapse, excitatory and inhibitory neurotransmission, presents fimbria-CA3 synapse preparation as an excellent model for dissecting the possible mechanisms involved in Aβ action on different septohippocampal neurotransmission systems.

A biphasic (glutamatergic, non-NMDA, and GABAergic, GABA_A_) response in CA3 region after fimbria stimulation (Schneiderman et al., 1992) has previously been reported. However, we found a complex synaptic response comprising three phases: an ionotropic glutamatergic EPSP (Huh et al., 2010) followed by two IPSPs, early (GABA_A_), and late (GABA_B_). This complex response has also been described in pyramidal neurons of basolateral amygdaloid nucleus (Washburn and Moises, 1992a), in CA3 pyramidal cells after hilus and mossy fibers stimulation (Malouf et al., 1990; Scanziani et al., 1991). In our study, the inhibitory feedback seems to be mediated by activation of GABAergic interneurons and depends on glutamatergic activation, since the block of EPSP also eliminated both IPSPs. The loss of GABA_A_ inhibition led to an increase in EPSP and late IPSP amplitudes whereas complete GABAergic component block induced an epileptic-like response mainly mediated by non-NMDA receptors, although NMDA antagonist reduced the response partially. Very similar results have been shown in neurons from CA3 hippocampal region (Scanziani et al., 1991) or frontal cortex (Sutor and Luhmann, 1998) possibly caused by an increased excitation of interneurons arising from disinhibited excitatory neurons, and disinhibition of interneurons due to block of GABA_A_ receptors on the inhibitory cell. This machinery has been suggested as a self-protective mechanism for the control of recurrent activity when an imbalance of the system occurs (Scanziani et al., 1991; Sutor and Luhmann, 1998).

On the other hand, although it has been reported that cholinergic septal neurons innervate pyramidal CA3 neurons and interneurons (Widmer et al., 2006), it is also known that cholinergic axons must be activated by train stimulation (>30 Hz) (Washburn and Moises, 1992b; Faber and Sah, 2002; Navarro-Lopez et al., 2004). It can therefore be suggested that cholinergic axons projecting onto CA3 neurons were not activated during single stimulation of the fimbria.

### Aβ_25–35_ effects on the complex fimbria-CA3 synaptic response

In the present study, pharmacological characterization of the complex septohippocampal synaptic response revealed the glutamatergic nature of the EPSP. Glutamatergic septohippocampal neurons (Colom et al., 2005; Huh et al., 2010) have shown spontaneous firing at *theta* frequencies and Aβ increases this frequency (Leao et al., 2012), suggesting Aβ to be likely to impair septohippocampal excitatory and network activity through more than one mechanism.

We found Aβ_25–35_ to increase the EPSP. This result may be explained because excitatory response is strongly braked by the GABA_B_ activity (Otmakhova and Lisman, 2004; Chen and Johnston, 2005), and Aβ_25–35_ diminished late IPSP. Since late IPSP was generated by GABA_B_ receptors stimulation, an Aβ_25–35_ effect on such receptors, on its intracellular signaling mechanism, or on its final effector, GirK channels, could be hypothesized. Assuming that the early GABA_A_ component is not affected by Aβ_25–35_, the reduction of GABA_B_ component would not be due to an inhibition of GABA release or other presynaptic mechanism. Together with Ri increase and membrane depolarization, our results lead to a reduction of the conductance of potassium channels coupled to GABA_B_ receptor.

Binding studies in postmortem AD patients have shown a reduction in GABA receptors density in the hippocampus (Chu et al., 1987). More recently, the 17A polymerase has been described to be responsible for generating alternative splicing of GABA_B2_ subunit in AD patients (Massone et al., 2011). This modification affects intracellular signaling pathway and activation of GirK channels, and is also associated with an increased secretion of Aβ, suggesting a relationship between the metabolism of APP protein and dysfunction in the GABA_B_ receptor signaling.

### Aβ_25–35_ action on GirK channels

When fimbria is stimulated, GABAergic interneurons activate GABA_B_ receptors of CA3 pyramidal cells; Aβ_25–35_ behaves as a selective antagonist and reduces late IPSP. However, the GABA_B_ system has a very low tonic activity in basal conditions, and only when the system is activated pharmacologically or by afferent stimulation, the antagonist effect becomes obvious (Bowery and Smart, 2006) as occurs, for example, in chronic pain (Malcangio and Bowery, 1994). For this reason, blocking GABA_B_ receptors induces little effects on membrane properties, i.e., membrane potential or Ri (Lambert et al., 1989; Emri et al., 1996), so GABA_B_ receptor antagonism would not explain all the effects induced by Aβ_25–35_.

Another aspect to consider is that both, GABA_B_ receptors and GirK channels are coupled and co-expressed in the postsynaptic membrane of CA3 pyramidal neurons (Luscher et al., 1997; Kulik et al., 2003; Lujan et al., 2009) conforming an oligomeric stable molecular complex (Lujan et al., 2009; Ciruela et al., 2010). Therefore, Aβ_25–35_ action on the membrane should include the effector coupled to GABA_B_ receptor. GirK channels exhibit a tonic basal activity, even without receptor signaling, due to their direct binding to the Gα subunit of G proteins (Lujan et al., 2009). Hence, administration of GirK channel selective antagonist (tertiapin-Q) simulates all the effects of Aβ_25–35_ on the postsynaptic membrane, i.e., late IPSP reduction, Ri increase and membrane depolarization, even in the presence of GirK and GABA_B_ agonists. Furthermore, Aβ_25–35_ was found to be unable to generate an additional significant increase in Ri after pharmacological blocking of GirK channels. These results not only suggest that GirK channels are functional in the basal state, but also that Aβ_25–35_ action seems to be more evident when these GirK channels are activated.

GirK channels activity alteration may have multiple implications for synaptic activity and neuronal network function. Numerous studies have emphasized its role in several pathological processes in the nervous system such as epilepsy, pain, addiction, Parkinson or Down syndrome (Luscher and Slesinger, 2010). Deletion studies of GirK channels have revealed their role in learning and memory processes. GIRK4 knock-out mice exhibited impaired performance in spatial learning and memory test (Wickman et al., 2000). Moreover, mutations in GIRK2 subunit reduced LTP and increased LTD in hippocampus (Sago et al., 1998; Siarey et al., 1999; Luscher and Slesinger, 2010) and it is especially relevant in Down syndrome, where cerebral Aβ accumulation is greatly accelerated and leads to invariant early-onset AD neuropathology (Lott and Head, 2005; Moncaster et al., 2010; Cooper et al., 2012).

The present study proposes a putative synaptic mechanism for neural network hyperactivity, which is considered as an early event in AD pathogenesis and is associated with early Aβ deposition in non-demented humans with or without mild cognitive impairment (Sperling et al., 2009). An alteration in GirK channel conductance of pyramidal CA3 neurons might underlie this hyperactivity and the impaired inhibition which has been related to network dysfunction and alteration of rhythm generation required for information processing and memory storage in the septohippocampal system (Palop et al., 2007; Palop and Mucke, 2010; Villette et al., 2010, 2012; Rubio et al., 2012; Verret et al., 2012). Our data could be in accordance to the notion that reducing network hyperactivity would have beneficial effects on cognitive functions (Palop and Mucke, 2010; Verret et al., 2012). Subsequently, the present work shows GirK channel as a new target to study Aβ pathophysiology in early and mild cognitive impairment in AD. Since cholinergic or glutamatergic treatments in AD have shown limited success, therapies combining modulators of different neurotransmission systems seem to be a more promising tool for the treatment, and overall prevention, of this dementia.

## Conflict of interest statement

The authors declare that the research was conducted in the absence of any commercial or financial relationships that could be construed as a potential conflict of interest.
